# Control of evolution of porous copper-based metal–organic materials for electroreduction of CO_2_ to multi-carbon products†

**DOI:** 10.1039/d3ma00033h

**Published:** 2023-04-05

**Authors:** Lili Li, Lutong Shan, Alena M. Sheveleva, Meng He, Yujie Ma, Yiqi Zhou, Marek Nikiel, Laura Lopez-Odriozola, Louise S. Natrajan, Eric J. L McInnes, Martin Schröder, Sihai Yang, Floriana Tuna

**Affiliations:** a Department of Chemistry, University of Manchester Manchester M13 9PL UK M.Schroder@manchester.ac.uk Sihai.Yang@manchester.ac.uk Floriana.Tuna@manchester.ac.uk; b Photon Science Institute, University of Manchester Manchester M13 9PL UK; c Institute for Advanced Materials and Technology, University of Science and Technology Beijing Beijing 100083 China; d Department of Materials, University of Manchester Manchester M13 9PL UK; e National Graphene Institute, University of Manchester M13 9PL UK

## Abstract

Electrochemcial reduction of CO_2_ to multi-carbon (C_2+_) products is an important but challenging task. Here, we report the control of structural evolution of two porous Cu(ii)-based materials (HKUST-1 and CuMOP, MOP = metal–organic polyhedra) under electrochemical conditions by adsorption of 7,7,8,8-tetracyanoquinodimethane (TNCQ) as an additional electron acceptor. The formation of Cu(i) and Cu(0) species during the structural evolution has been confirmed and analysed by powder X-ray diffraction, and by EPR, Raman, XPS, IR and UV-vis spectroscopies. An electrode decorated with evolved TCNQ@CuMOP shows a selectivity of 68% for C_2+_ products with a total current density of 268 mA cm^−2^ and faradaic efficiency of 37% for electrochemcial reduction of CO_2_ in 1 M aqueous KOH electrolyte at −2.27 V *vs.* RHE (reversible hydrogen electrode). *In situ* electron paramagnetic resonance spectroscopy reveals the presence of carbon-centred radicals as key reaction intermediates. This study demonstrates the positive impact of additional electron acceptors on the structural evolution of Cu(ii)-based porous materials to promote the electroreduction of CO_2_ to C_2+_ products.

## Abstract 2

Formation of multi-carbon (C_2+_) products by electrochemical reduction of CO_2_ using two porous Cu(ii)-based materials (HKUST-1 and CuMOP, MOP = metal–organic polyhedra) under electrochemical conditions in the presence of TNCQ.

## Graphical abstract

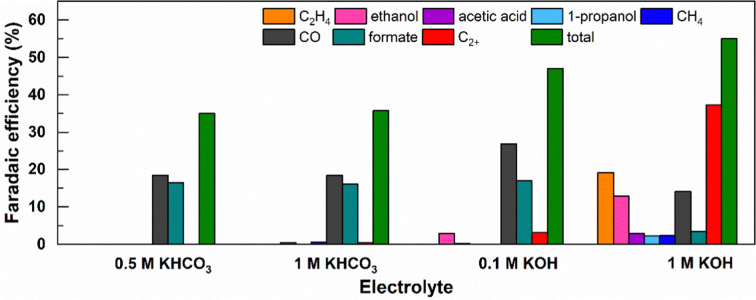

## Introduction

Electrochemical CO_2_ reduction reaction (CO_2_RR) using renewable electricity enables the sustainable synthesis of feedstock chemicals.^1,2^ Compared with C_1_ products (*e.g.*, CO, HCOOH), multi-carbon (C_2+_) products (*e.g.*, C_2_H_4_, C_2_H_5_OH) are desirable because of their higher volumetric energy density and commercial value,^3,4^ but this is a highly challenging target. Copper-based electrocatalysts can show excellent activity to convert CO_2_ to C_2+_ products *via* ˙C-based radicals which can couple to afford C_2+_ products.^5–8^ Porous metal–organic framework (MOF) materials have emerged as efficient catalysts for CO_2_RR owing to the presence of atomically dispersed metal sites, and their porous structure allows adsorptive binding of CO_2_.^9–12^ Several Cu(ii)-based MOFs have been tested for electrochemical CO_2_RR, but they generally suffer from limited electrical conductivity and hydrolytic stability.^13–15^ Structural evolution of Cu(ii)-MOFs to active catalysts such as Cu, Cu_2_O, Cu@Cu_*x*_O under electrochemical conditions often results in improved conductivity and stability, and has attracted much interest.^16–22^ In particular, evolved Cu(i) species can facilitate the coupling of ˙CO radical intermediates and thus promote the production of C_2+_ products.^22,23^ The control of the structural evolution of Cu(ii)-MOFs is key to produce C_2+_ products in CO_2_RR.

7,7,8,8-Tetracyanoquinodimethane (TCNQ) is an excellent electron acceptor and can be reduced readily to TCNQ˙^−^ and TCNQ^2−^.^24,25^ It has been reported that adsorbed TCNQ molecules can replace the axially coordinated water molecules of the [Cu^II^_2_(OOCR)_4_] paddlewheel in HKUST-1 to form a network of inter-connected [Cu^II^_2_(OOCR)_4_] paddlewheels, resulting in a significant increase in electrical conductivity.^26^ Here, we report the investigation of the impact of TCNQ on the structural evolution of Cu(ii)-based porous materials during the electrochemical CO_2_RR process. In addition to HKUST-1, another highly porous Cu(ii)-based metal–organic polyhedra material, CuMOP,^27^ was chosen for this study because it exhibits mesopores formed from the packing of coordination cages and these might promote the capture of bulky TCNQ molecules within the structure. TCNQ was introduced as the guest species to tune the conductivity of HKUST-1 and CuMOP, and the materials HKUST-1, CuMOP, TCNQ@HKUST-1 and TCNQ@CuMOP were used as precursors to study the evolution of these systems to active catalysts for CO_2_RR in a flow-cell. Powder X-ray diffraction (PXRD), infrared (IR), Raman, ultraviolet-visible (UV-vis), electron paramagnetic resonance (EPR) and X-ray photoelectron spectroscopy (XPS), and scanning electron microscopy (SEM) have been employed to characterise the structural evolution of these materials under electrochemical conditions. The evolved materials are denoted as HKUST-1-p, CuMOP-p, TCNQ@HKUST-1-p and TCNQ@CuMOP-p (p = potential). Significantly, the electrode decorated with evolved TCNQ@CuMOP-p shows a selectivity of 68% for C_2+_ products with a C_2+_ current density of 100 mA cm^−2^ and faradaic efficiency of multi-carbon products (FE_C_2+__) of 37% in 1.0 M KOH electrolyte. *In situ* EPR spectroscopy using the spin trap, 5,5-dimethyl-1-pyrroline-N-oxide (DMPO), confirms the presence of carbon-centred radicals as the main reaction intermediates. This study demonstrates the positive impact of TCNQ on the structural evolution of Cu(ii)-based porous materials for electrocatalytic CO_2_RR.

## Results and discussion

### Characterization of adsorption of TCNQ

**Fig. 1:**
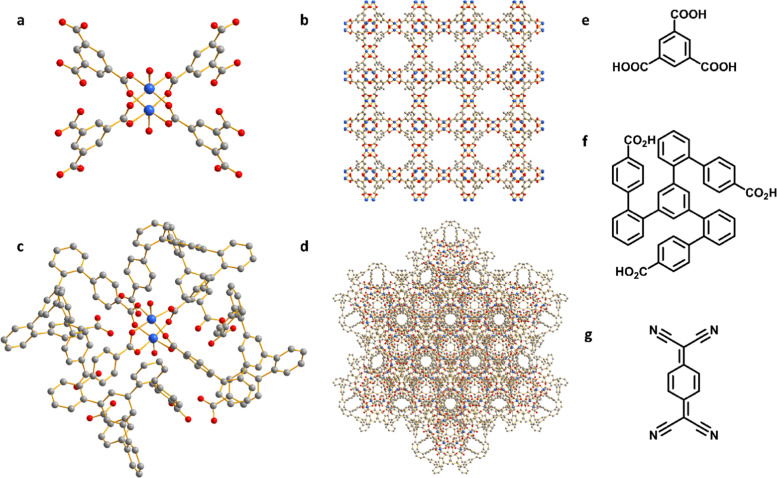
Views of (a) the [Cu_2_(OOCR)_4_] paddlewheel in HKUST-1; (b) structure of HKUST-1; (c) the [Cu_2_(OOCR)_4_] paddlewheel in CuMOP; (d) structure of CuMOP; (e) benzene-1,3,5-tricaroxylic acid used in HKUST-1; (f) ligand used in CuMOP; (g) TCNQ. Cu: blue; O: red; C: grey; hydrogen atoms are omitted for clarity.

**Fig. 2:**
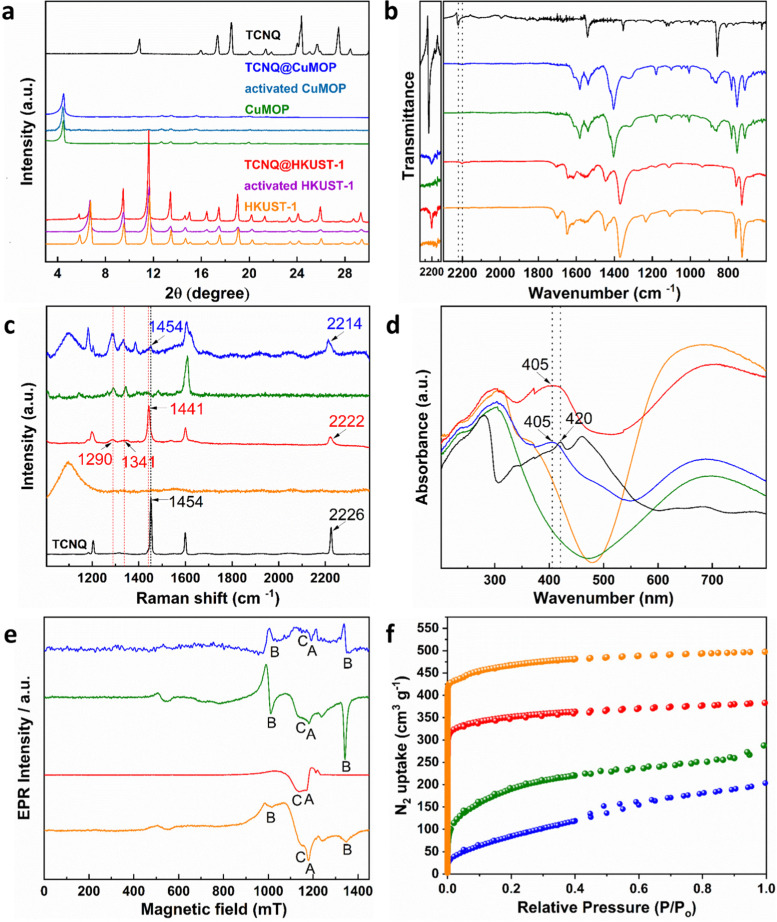
Characterisation of adsorption of TCNQ into HKUST-1 and CuMOP. Views of (a) PXRD patterns; (b) IR spectra; (c) Raman spectra; (d) UV-vis spectra; (e) Q-band EPR spectra (feature A is due to monomeric Cu(ii) monomer centers, features B are due to the intra-binuclear interaction within [Cu_2_(OOCR)_4_] paddlewheels, and feature C is due to the inter-binuclear spin-exchange between [Cu_2_(OOCR)_4_] paddlewheel centers; HKUST-1 and TCNQ@HKUST-1 at 100 K, CuMOP and TCNQ@CuMOP at 293 K. (f) N_2_ adsorption/desorption isotherms at 77 K. (HKUST-1: orange, TCNQ@HKUST-1: red, CuMOP: green, TCNQ@CuMOP: blue, TCNQ: black).

HKUST-1, [Cu_3_(OH_2_)_3_(C_9_H_3_O_6_)_2_], is constructed by [Cu_2_(OOCR)_4_] paddlewheels bridged by benzene-1,3,5-tricarboxylate ligands to afford an open framework structure (Fig. 1).^28^ In contrast, CuMOP, [Cu_12_(C_45_H_27_O_6_)_8_(H_2_O)_9_ ⊃ (H_2_O)_2_] packs to form two types of intermolecular voids between spherical cages, in addition to the internal cavities within these cages. The larger intermolecular octahedral cavities are 3336 Å^3^ per unit cell in size with the smaller intermolecular tetrahedral cavities having a volume of 114 Å^3^ per unit cell. The cage cavity has an internal volume of 272 Å^3^ (Fig. 1).^27^ The phase purity of these materials was confirmed by PXRD (Fig. 2a), and the materials were activated by heating at 180 °C for 3 h. The activated materials were then soaked in a saturated solution of TCNQ in CH_2_Cl_2_ at room temperature for 3 days to allow full adsorption of TCNQ.^26^ The solid was then isolated *via* filtration and dried at 65 °C for 24 h to remove adsorbed CH_2_Cl_2_. PXRD patterns confirm the retention of the crystal structure of HKUST-1 and CuMOP upon adsorption of TCNQ. IR spectra show that the C≡N stretching mode of TCNQ is shifted from 2222 to 2200 cm^−1^ (corresponding to a charge transfer of ∼0.4 e^−^ between the framework and TCNQ) upon adsorption into HKUST-1 and CuMOP (Fig. 2b). The Raman spectrum of TCNQ@HKUST-1 confirms that the C=C stretching vibrational modes of TCNQ, normally at 1454 to 1441 cm^−1^, are shifted to 1341 and 1290 cm^−1^ (Fig. 2c), consistent with partial charge-transfer between HKUST-1 and TCNQ and indicating that adsorbed TCNQ molecules may well be interacting with the Cu(ii) sites.^29^ In comparison, TCNQ@CuMOP exhibits little change to the C=C stretching mode and the C≡N stretching mode is only shifted from 2226 to 2214 cm^−1^ (Fig. 2c), suggesting that TCNQ molecules primarily reside in the large voids rather than in the metal-ligand cages.

The UV-vis spectra of TCNQ@HKUST-1 and TCNQ@CuMOP show a peak at 405 nm (Fig. 2d), lower than that observed for the neutral TCNQ molecule (420 nm), further confirming the adsorption of TCNQ. Continuous-wave (CW) Q-band EPR spectroscopy was used to characterize TCNQ@HKUST-1 and TCNQ@CuMOP. In a [Cu^II^_2_(OOCR)_4_] paddlewheel, the two neighbouring Cu(ii) (*s* = 1/2) ions couple antiferromagnetically to give an *S* = 0 ground state (EPR silent) and an excited *S* = 1 state. To observe the EPR signal for the [Cu^II^_2_(OOCR)_4_] paddlewheel, variable temperature CW Q-band EPR experiments were conducted (Fig. S2, ESI†). The EPR signal of excited *S* = 1 triplet state of [Cu^II^_2_(OOCR)_4_] paddlewheels in HKUST-1 and CuMOP can be seen at 100 K and 293 K, respectively.^30^ Therefore, the Q-band EPR spectra of TCNQ@HKUST-1 at 100 K and of TCNQ@CuMOP at 293 K were chosen to compare with the un-modified parent materials. The EPR spectrum of HKUST-1 contains three types of EPR signals:^30,31^ (i) a sharp signal due to monomeric, *s* = 1/2 Cu(ii) sites (feature A), which in [Cu^II^_2_(OOCR)_4_] paddlewheel-MOFs has been attributed to extra-framework uncoupled Cu(ii) centres formed during the synthesis; (ii) excited *S* = 1 intra-binuclear triplet state of the [Cu^II^_2_(OOCR)_4_] paddlewheel (feature B), spread over a wide field range due to a zero-field splitting |*D*| of *ca.* 0.325 cm^−1^ (Table S1 and Fig. S3, ESI†), and (iii) a broad isotropic feature (feature C) arising from inter-dimer exchange (*J*′ ≈ 1 cm^−1^; Table S1, ESI†) between neighbouring *S* = 1 populated [Cu^II^_2_(OOCR)_4_] paddlewheels (Fig. 2e). Only features A and C are observed in TCNQ@HKUST-1 with the latter being dominant (Fig. S3b (ESI†) and Fig. 2e). This suggests that adsorbed TCNQ molecules enhance the inter-paddlewheel exchange in TCNQ@HKUST-1. In contrast, the EPR signal of *S* = 1 [Cu^II^_2_(OOCR)_4_] paddlewheels is observed in both CuMOP and TCNQ@CuMOP (Fig. 2e and Fig. S3, ESI†), indicating that the adsorption of TCNQ in the voids has little impact on the [Cu^II^_2_(OOCR)_4_] paddlewheel signature. This is consistent with the smaller shifts in the IR spectrum for TCNQ for this material compared with HKUST-1. N_2_ adsorption isotherms at 77 K show a reduction in the surface area of HKUST-1 (from 1449 to 1083 m^2^ g^−1^) and CuMOP (from 664 to 342 m^2^ g^−1^) upon adsorption of TCNQ (Fig. 2f) reflecting occupation of pores by TCNQ.

### Characterisation of structural evolution

**Fig. 3:**
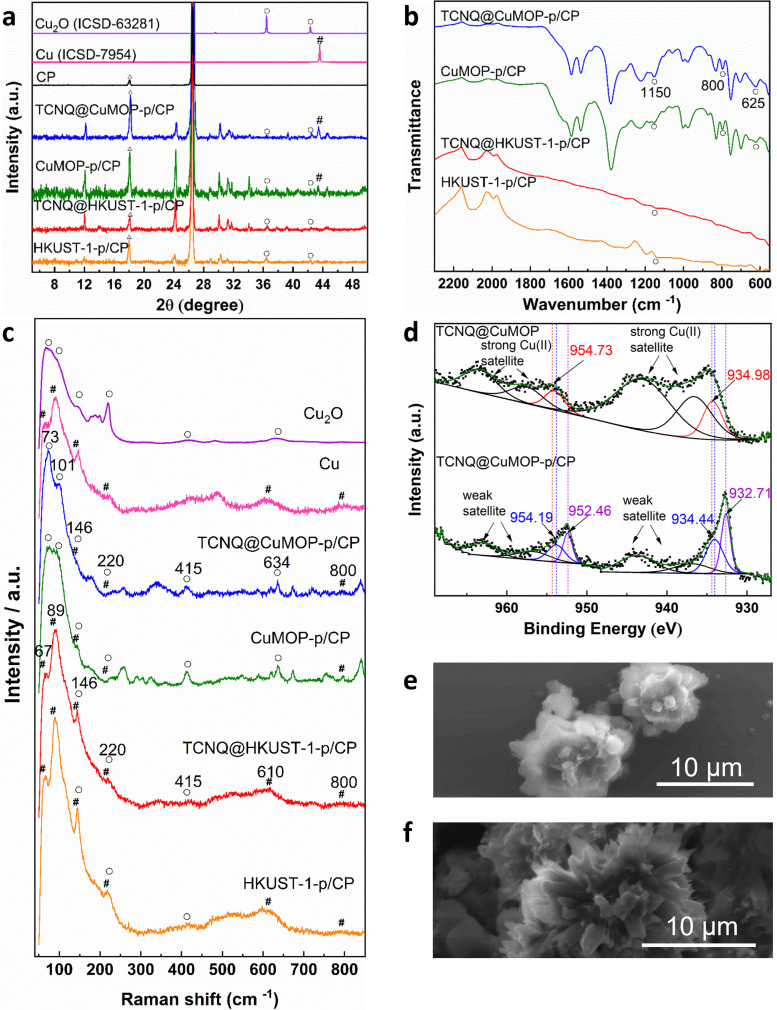
Spectroscopic and morphological characterization of the evolution of working electrodes. (a) PXRD patterns; (b) IR spectra; (c) Raman spectra; (d) Cu 2p region XPS spectra; SEM images of (e) TCNQ@CuMOP and (f) TCNQ@CuMOP-p/CP (Δ refers to CP, ○ refers to Cu_2_O, # refers to Cu).

Aqueous solutions of KHCO_3_ and KOH (0.1–1.0 M) were tested as electrolyte in a flow cell to optimise the electrochemical CO_2_RR at −2.27 V *vs.* RHE. A solution of 1 M KOH showed the best performance and was therefore used in this study (Fig. S4–S8, ESI†).The PXRD patterns confirm the structural evolution of both pristine and TCNQ-loaded materials upon applying a potential of −2.27 V *vs.* RHE for 10 min, and the resulting working electrodes are denoted as HKUST-1-p/CP (CP = carbon paper), TCNQ@HKUST-1-p/CP, CuMOP-p/CP and TCNQ@CuMOP-p/CP. Taking TCNQ@CuMOP-p/CP as an example, the PXRD pattern confirms the formation of Cu_2_O and Cu (Fig. 3a and Fig. S9). IR, Raman and XPS spectra also confirm the formation of Cu_2_O and Cu in TCNQ@CuMOP-p (Fig. 3b–d). Compared with TCNQ@CuMOP, new IR bands at 1150, 800 and 625 cm^−1^ are observed for TCNQ@CuMOP-p, confirming the formation of Cu_2_O (Fig. 3b and Fig. S10d, ESI†).^32,33^ Raman spectra of TCNQ@CuMOP-p also show the characteristic peaks at 73, 101, 146, 220, 415, 634 and 800 cm^−1^ assigned to Cu_2_O and Cu, with the former being the main phase (Fig. 3c).^34–36^ XPS spectra show two peaks for Cu 2p at BE = 954.73 and 934.98 eV in TCNQ@CuMOP corresponding to Cu(ii) centers (Fig. 3d).^37^ In TCNQ@CuMOP-p, two sets of Cu 2p peaks are observed: the peaks at BE = 954.19 and 934.44 eV correspond to new Cu(ii) species surrounded by disordered microenvironments resulting from the structural evolution of TCNQ@CuMOP. The peaks at BE = 952.46 and 932.71 eV correspond to Cu(i) species with a minor amount of Cu(0). Similar PXRD, IR and Raman results are observed for CuMOP-p (Fig. 3a–c and Fig. S9 and S10, ESI†). Interestingly, the XPS spectra of CuMOP-p show two sets of Cu 2p peaks (Fig. S11 and S12): the peaks at BE = 952.8 and 933.0 eV correspond to Cu(i) and the peaks at BE = 950.9 and 931.2 eV correspond to Cu(0). This result confirms that adsorbed TCNQ in CuMOP may serve as an electron acceptor thus controlling the formation of Cu(i) over Cu(0) species. PXRD, IR and Raman results for HKUST-1-p and TCNQ@HKUST-1-p also confirm the formation of both Cu_2_O and Cu(0), and Raman spectra show strong signals for Cu(0) in both HKUST-1-p and TCNQ@HKUST-1-p. Overall, TCNQ@CuMOP-p shows higher selectivity for the formation of Cu_2_O over Cu(0) species compared with TCNQ@HKUST-1-p, and this is likely due to (i) the large voids of CuMOP that allow better adsorption of TCNQ and (ii) the hindered charge transport in CuMOP owing to its isolated cages during evolution. SEM images confirm changes from cubic to needle-like morphology for HKUST-1-p and TCNQ@HKUST-1-p and flower-like morphology for CuMOP-p and TCNQ@CuMOP-p upon structural evolution (Fig. 3e and f and Fig. S13 and S14, ESI†).

### Electrochemical CO_2_RR

**Fig. 4:**
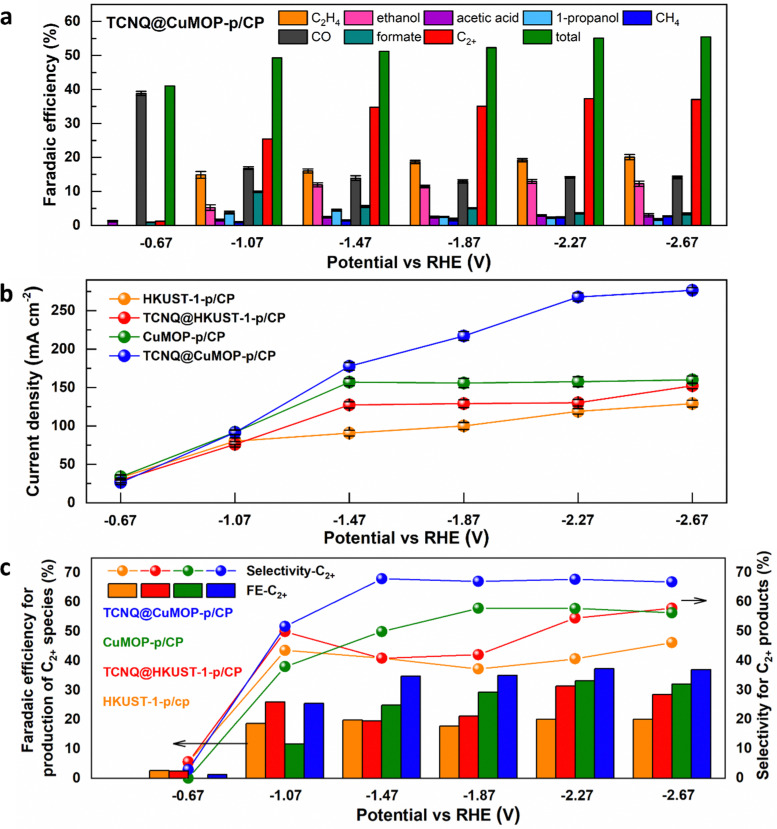
(a) Catalytic performance of the TCNQ@CuMOP-p/CP electrode for CO_2_RR at different potentials. (b) Comparation of the total current density over different electrodes for CO_2_RR at different potentials. (c) Faradaic efficiency (FE) and selectivity to C_2+_ products over different electrodes for CO_2_RR at different potentials.

HKUST-1-p/CP, TCNQ@HKUST-1-p/CP, CuMOP-p/CP, and TCNQ@CuMOP-p/CP were used as working electrodes to conduct CO_2_RR as a function of potential in aqueous 1 M KOH. Ethylene, ethanol, acetic acid, 1-propanol, methane, CO, and formate are all detected as products (Fig. 4a and Fig. S15–S17, ESI†). In contrast, no carbon-based product was detected using TCNQ/CP as a catalyst. Of the four working electrodes, TCNQ@CuMOP-p/CP shows the best catalytic performance with FE_C_2+__ of 37% (with a FE_total_ of 55%, thus a selectivity to C_2+_ products of 68%), a total current density of 268 mA cm^−2^, and C_2+_ current density of 100 mA cm^−2^ at −2.27 V *vs.* RHE (Fig. 4a–c). In comparison, the FE_C_2+__, current density and the selectivity for C_2+_ products over CuMOP-p/CP (33%, 52.2 mA cm^−2^, 58%), TCNQ@HKUST-1-p/CP (31%, 40.9 mA cm^−2^, 55%) and HKUST-1-p/CP (20%, 24.0 mA cm^−2^, 41%) electrodes are lower (Fig. 4c). Importantly, the catalytic performance of TCNQ@CuMOP-p/CP is comparable with the leading Cu(ii)-MOF-derived catalysts studied for CO_2_RR (Table S3, ESI†).

**Fig. 5:**
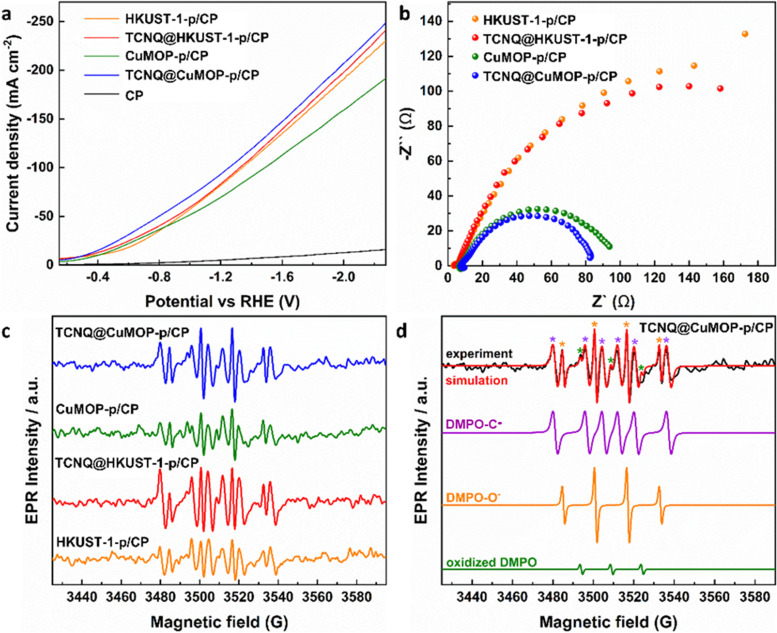
(a) Linear sweep voltammetry (LSV) in CO_2_-saturated electrolyte. (b) EIS spectra. (c) EPR spectra of electrolyte aliquots taken with different catalysts after electrolysis for 30 min. (d) EPR spectra of spin adducts of free radicals. The complete set of parameters for simulations are given in Tables S2 and S3 (ESI†).

Linear sweep voltammetry (LSV) was used to evaluate the electrochemical response of these evolved working electrodes to reactivity with CO_2_ (Fig. 5a). The reductive current of all four decorated electrodes is significantly higher than that of bare CP, confirming the activity of the MOF-derived catalysts towards CO_2_RR. The reductive current of TCNQ@CuMOP-p/CP is much higher than that of CuMOP/CP when the potential is more negative than −0.4 V *vs.* RHE. The reductive current of TCNQ@HKUST-1-p/CP is also higher than that of HKUST-1-p/CP when the potential is more negative than −1.4 V *vs.* RHE. Electrochemical impedance spectroscopy (EIS) reveals the charge-transfer resistance (*R*_ct_) of HKUST-1-p/CP (340 Ω cm^2^), TCNQ@HKUST-1-p/CP (300 Ω cm^2^), CuMOP-p/CP (100 Ω cm^2^), and TCNQ@CuMOP-p/CP (87 Ω cm^2^) in the electrolyte (Fig. 5b). These results suggest that adsorption of TCNQ has a positive impact on the conductivity of these evolved catalysts. The high conductivity of TCNQ@CuMOP-p/CP promotes its high FE_C_2+__ and selectivity for C_2+_ products as observed in the electrocatalysis tests.

### EPR Spectroscopy

EPR spectroscopy was employed to monitor and characterise the intermediate radicals produced during the electroreduction process. EPR spectra were measured for aliquots of electrolyte solution taken at time intervals of 10 min during reduction of CO_2_ at −2.27 V *vs.* RHE over all four electrodes. DMPO was used as a spin trapping agent to identify the short-lived radicals during CO_2_RR.^38^ Characteristic spectra of DMPO-adduct radicals based upon O and C radicals, DMPO-O and DMPO-C, respectively, were observed for all four electrodes (Fig. 5c and d) and the signal intensity remains stable during the CO_2_RR for all electrodes (Fig. S18, ESI†). However, within a given timeframe, the intensity of the EPR signal is significantly stronger for TCNQ-modified working electrodes compared with HKUST-1-p/CP and CuMOP-p/CP, consistent with their improved catalytic performance upon adsorption of TCNQ (Fig. 5c).

## Conclusion

The design and control of the structural evolution of porous materials during electrochemical CO_2_RR represents an important route to new electrocatalysts. We report the control of structural evolution of HKUST-1 and CuMOP by adsorption of TCNQ as an additional electron acceptor to drive the reduction of Cu(ii) sites to Cu(i) species, which promote the coupling of ˙C radical intermediates to yield C_2+_ products. PXRD, IR, Raman, XPS and EPR spectroscopy have been applied to investigate the evolution process and reveal the formation of Cu(i) and Cu(0) products. TCNQ@CuMOP-p/CP shows a high selectivity of 68% for C_2+_ products with FE_C_2+__ of 37% and a total current density of 268 mA cm^−2^ at −2.27 V *vs.* RHE, comparable with the leading Cu(ii)-MOF-derived catalysts studied for CO_2_RR, with the remaining FE reflecting competing production of H_2_. EPR spectroscopy, coupled with a spin trap experiments, confirms the formation of carbon-centred radicals as the key reaction intermediate in this system. Interestingly, oxalic acid was not observed as a product to any great extent reflecting the aqueous conditions employed in the current study. Oxalic acid generated by coupling of ˙CO_2_ radicals is more frequently observed in reductions in non-aqueous media.^39–43^ This study provides a facile pathway to control the structural evolution of porous materials by introducing additional electron acceptors to promote their activity for efficient electrochemical conversion of CO_2_ to multi-carbon products.

## Conflicts of interest

The authors declare no competing financial interest.

## Supplementary Material

MA-004-D3MA00033H-s001
