# Supplementary material for: Control of evolution of porous copper-based metal–organic materials for electroreduction of CO2 to multi-carbon products
Source: Mater Adv. 2023 Apr 5;4(8):1941–8. doi: 10.1039/d3ma00033h (PMC10123487; doi:10.1039/d3ma00033h)
Supplement: MA-004-D3MA00033H-s001 [file MA-004-D3MA00033H-s001.pdf]

Electronic Supplementary Material (ESI) for Materials Advances.
This journal is © The Royal Society of Chemistry 2023

# Supporting Information

# Control of evolution of porous copper-based metal-organic materials for electroreduction of $CO_2$ to multi-carbon products

Lili Li[1], Lutong Shan[1], Alena M. Sheveleva[1,2], Meng He[1], Yujie Ma[1], Yiqi Zhou[3], Marek Nikiel[2,4,5], Laura Lopez-Odriozola[1], Louise S. Natrajan[1], Eric J. L McInnes[1,2], Martin Schröder[1]*, Sihai Yang[1]* and Floriana Tuna[1,2]*

1. Department of Chemistry, University of Manchester, Manchester, M13 9PL (UK)
M.Schroder@manchester.ac.uk; Sihai.Yang@manchester.ac.uk; Floriana.Tuna@manchester.ac.uk
2. Photon Science Institute, University of Manchester, Manchester, M13 9PL (UK)
3. Institute for Advanced Materials and Technology, University of Science and Technology Beijing, Beijing 100083 (China)
4. Department of Materials, University of Manchester, Manchester, M13 9PL (UK)
5. National Graphene Institute, University of Manchester, M13 9PL (UK)

**Experimental section**

**Materials.** $Cu(NO_3)_2{\cdot}3H_2O$ (>99%), benzene-1,3,5-tricarboxylic acid ($H_3BTC$) (>99%), tetracyanoquinodimethane (TCNQ), ethanol (EtOH) (>99%), dimethylformamide (DMF) (>99%), acetone (>99%), dichloromethane ($CH_2Cl_2$) (>99%), dimethyl sulfoxide (DMSO), 5,5-dimethyl-1-pyrroline-N-oxide (DMPO) and deuterium oxide ($D_2O$) were purchased from Sigma-Aldrich Co., UK. Carbon paper (CP) and polytetrafluoroethylene (PTFE) were purchased from Gaoss Union. KOH (99%), Nafion D521 solution (5% w/w in water and 1-propanol, ≥ 0.92 meq/g exchange capacity) and anion exchange membrane were purchased from Alfa Aesar Co., UK. $H_3L$ used for the synthesis of CuMOP was prepared according to our previously reported procedure.[1] All chemicals and reagents used in this study were used as received without further purification.

**Synthesis of HKUST-1.** A solution of $Cu(NO_3)_2{\cdot}3H_2O$ (7.8 g, 32.1 mmol) in $H_2O$ (90mL) was slowly added to a solution of $H_3BTC$ (2.04 g, 9.6 mmol) in EtOH (90 mL). After continuous stirring for 10 min at room temperature, 6 mL DMF was added to the solution. The vial was sealed and heated at 80 $^{o}C$ for 20 h. The resultant powder was collected and washed several times with DMF and acetone and dried.

**Synthesis of CuMOP.** CuMOP was synthesised according to our previously reported procedure.[1]

**Material characterisation.** PXRD data were collected from a Philips X'pert X-ray diffractometer (45 kV and 40 mA) using Cu- Kα radiation (λ = 1.5406 Å), and the scan speed was $1^{o}$/min. Fourier Transformed Infrared (FTIR) spectra were collected with Nicolet iS5 spectrometer, and Raman spectra were recorded on a Horiba XploRA Plus Raman microscope with a 638 nm laser and 1200 $mm^{-1}$ grating. Baseline correction was applied to all Raman spectra. Electron paramagnetic resonance (EPR) spectra of solid samples were recorded at different temperature in continuous wave mode on Bruker EMX spectrometers (Q-band, ca. 34 GHz), at mw power of ~0.63mW and modulation amplitude 10 G. EPR spectra of liquid samples was collected at X-band (9.85 GHz) on a Bruker Micro EPR spectrometer at room temperature with a microwave power of 6.325 mW and an modulation amplitude of 1 G. DMPO (200 mmol/L) was dissolved in Ar-degassed de-ionised water and used as a spin trap; a Bruker strong pitch (g = 2.0028) reference was used as a calibrator. Theoretical modelling of the spectra was performed with the EasySpin toolbox within Matlab.[2] The BET surface areas were obtained from $N_2$ adsorption/desorption isotherms recorded on a Micromeritics 3-Flex instrument at 77 K. X-ray photoelectron spectroscopy (XPS) analysis was performed using a Kratos Axis Ultra Hybrid spectrometer with monochromatized Al Kα X-ray source, using 20 eV energy pass for core levels spectra. C 1s electron at binding energy of 284.8 eV was used as a standard reference to calibrate the photoelectron energy shift. All the data analysis was

performed on the Casa XPS software (version: 2.3.22PR1.0). Peak deconvolution was performed with Tougaard type background and LA peak shape. The morphologies of the materials were measured by scanning electron microscopy (SEM) on a Quanta FEG 650.

**Electrochemical study.** All electrochemical experiments were carried out on a CHI 660E, USA electrochemical workstation with a flow cell. Carbon paper (CP) was used as the substrate for preparing working electrodes. The working electrodes HKUST-1/CP, TCNQ@HKUST-1/CP, CuMOP/CP and TCNQ@CuMOP/CP were prepared using the following procedure: 10 mg of HKUST-1, TCNQ@HKUST-1, CuMOP or TCNQ@CuMOP was suspended in 750 μL isopropanol and 250 μL $H_2O$ containing 100 μL Nafion D-521 dispersion (5 wt%). This was treated with ultrasound for 30 mins to form a homogeneous ink. 100 μL of the ink was spread onto the CP (1×1 cm$^2$) surface and dried at room temperature. To increase the hydrophobicity of the working electrode, PTFE was placed on the gas chamber side, just behind the catalyst/CP working electrode, to prevent the catholyte from entering the gas chamber.

The flow cell (Supplementary Figure 4) contains a hydrophobic and porous cathodic working electrode separating the gas and catholyte chambers. The cathode and anode are separated by an anion exchange membrane, and 1.0 M KOH solution was used as both catholyte and anolyte, and passed through the cathode and anode chambers separately. Ag/AgCl (in saturated KCl) was used as the reference electrode and Pt was the counter electrode. $CO_2$ (50 sccm) was passed over the porous working electrode, and then reduced over the catalyst into the cathodic section. After electrolysis, the liquid products were studied by $^1$H NMR spectroscopy and gas products were collected in a gasbag and analysed by Bruker Matrix MG5 FTIR spectrometer.

Electrochemical impedance spectroscopy (EIS) was recorded at −0.174 V vs RHE with an amplitude of 5.0 mV ($10^{-1}$ to $10^6$ Hz). The value for the resistance of charge transfer ($R_{ct}$) was obtained by fitting the EIS spectra using the Zview software (Version 3.5f, Scribner Associates, Inc). Linear sweep voltammetry (LSV) scans were conducted in $CO_2$ and Ar saturated catholyte.

**Quantitative analysis of products in liquid and gas phase.** All liquid products were quantified using $^1$H NMR spectroscopy. DMSO (1.0g) was dissolved in $H_2O$ (25 mL) and this solution used as the reference. After the $CO_2$RR, 100 μL of the as-prepared reference solution of DMSO in $H_2O$ was injected into the catholyte. Then 0.9 mL of catholyte was mixed with 0.1 mL $D_2O$, and around 0.7 mL of this solution was subsequently transferred into an NMR tube for measurement. Gas products were quantified by Bruker Matrix MG5 FTIR spectrometer The value of FE was calculated using the equation:

$$FE(\%) = \frac{n_{product} \times n_{electrons} \times F}{Q} \times 100\%$$

where $n_{product}$ is the amount of product (mol) from Bruker Matrix MG5 FTIR spectrometer or $^1H$ NMR spectroscopy (formic acid), $n_{electrons}$ is electron transfer number (both the production of $H_2$ and formate are two-electron processes), $F$ is the Faraday constant (96485 C $mol^{-1}$), and $Q$ is the total charge passed during the $CO_2RR$.

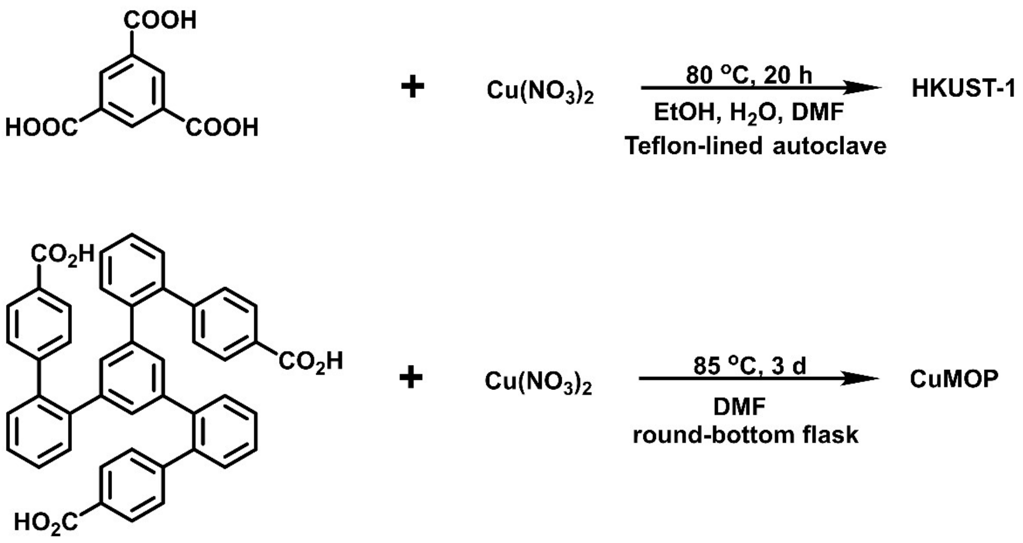


**Supplementary Figure 1.** Synthesis of HKUST-1 and CuMOP.

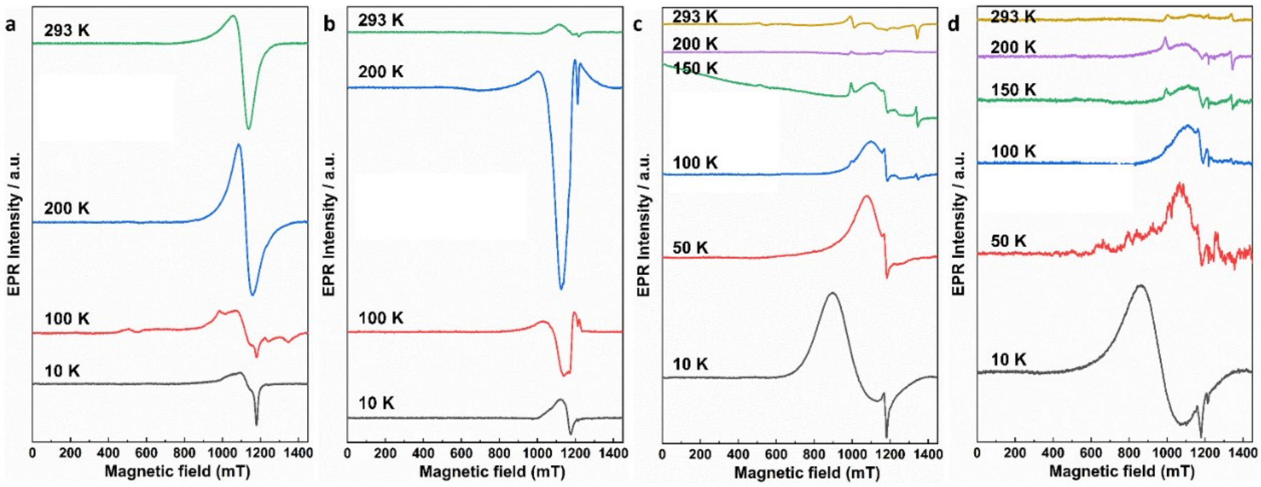


**Supplementary Figure 2.** Q-band EPR spectra at different temperatures. (a) HKUST-1, (b) TCNQ@HKUST-1, (c) CuMOP, and (d) TCNQ@CuMOP.

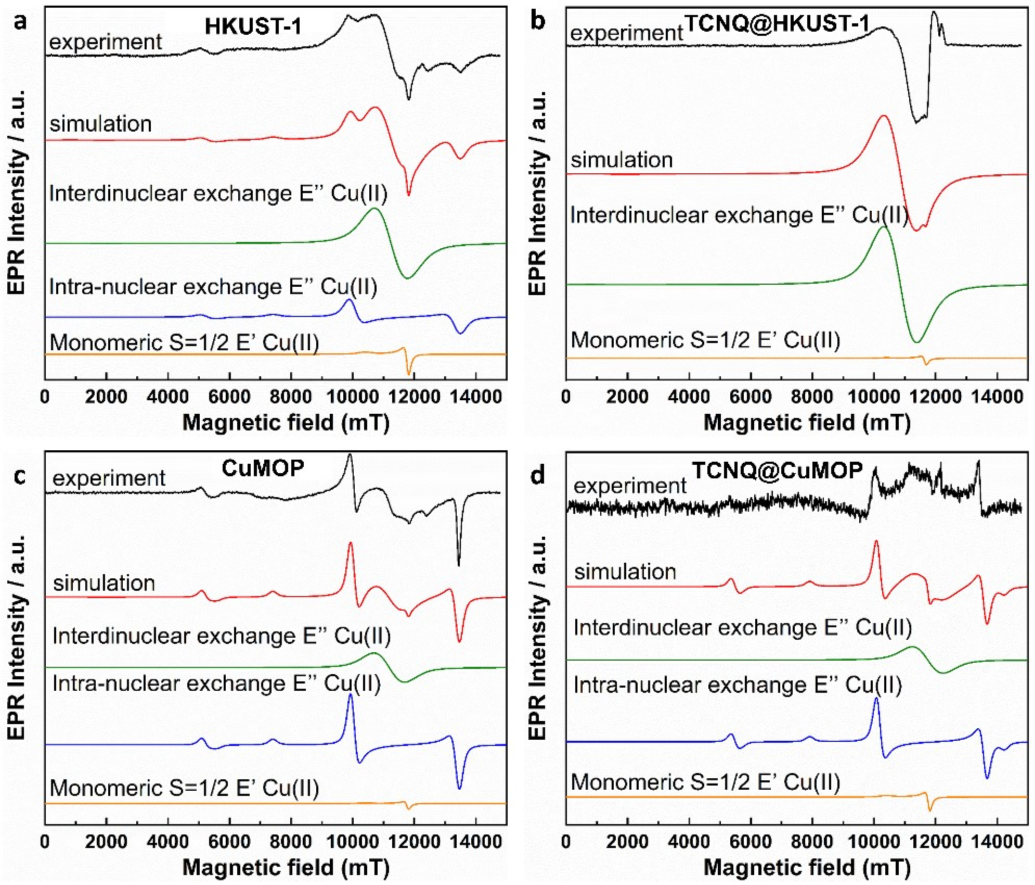


**Supplementary Figure 3.** Q-band EPR spectra and simulations. (a) HKUST-1, (b) TCNQ-HKUST-1, (c) CuMOP and (d) TCNQ@CuMOP. The experimental EPR spectra for HKUST-1 and TCNQ@HKUST-1 were collected at 100 K; for CuMOP and TCNQ@CuMOP they were collected at 293 K.

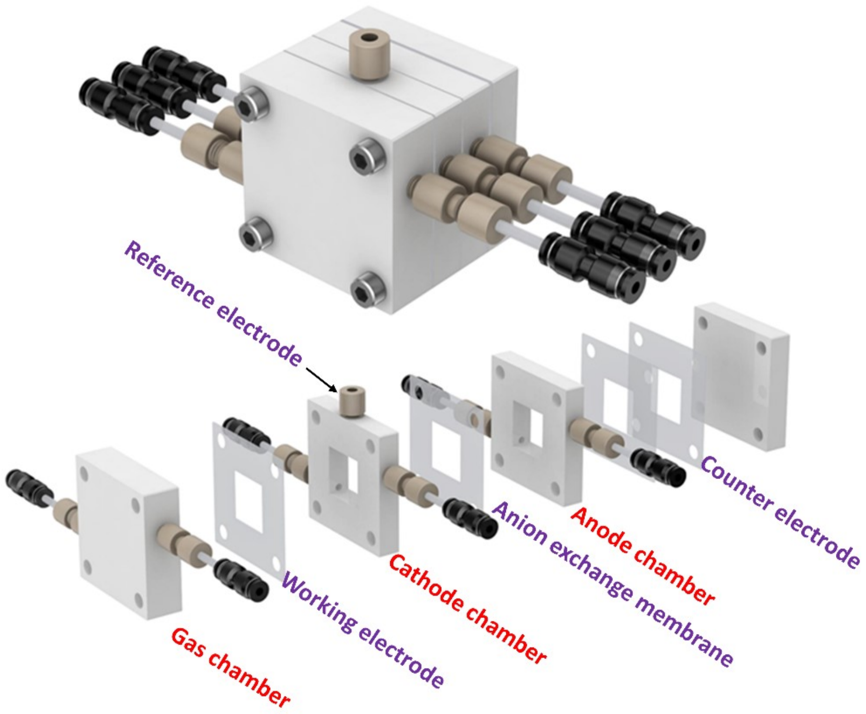


**Supplementary Figure 4.** View of the flow-cell used in this study.

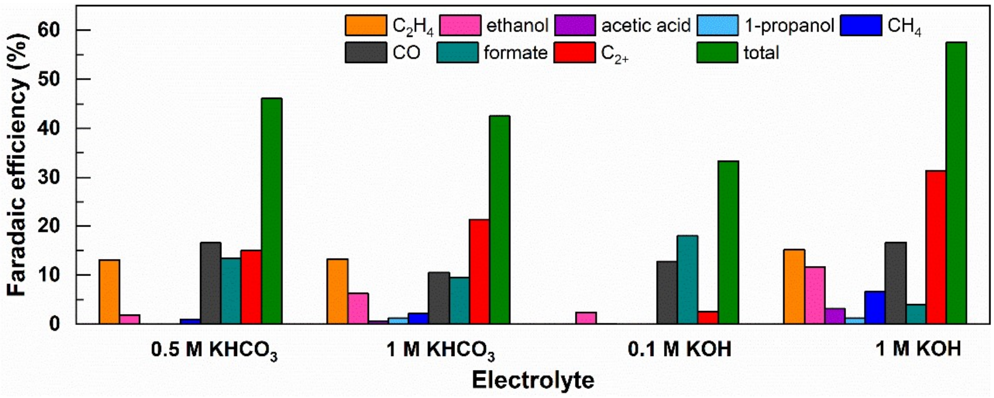


**Supplementary Figure 5.** Catalytic performance of TCNQ@HKUST-1-p/CP electrode at −2.27 V vs RHE for $CO_2$RR in different electrolytes.

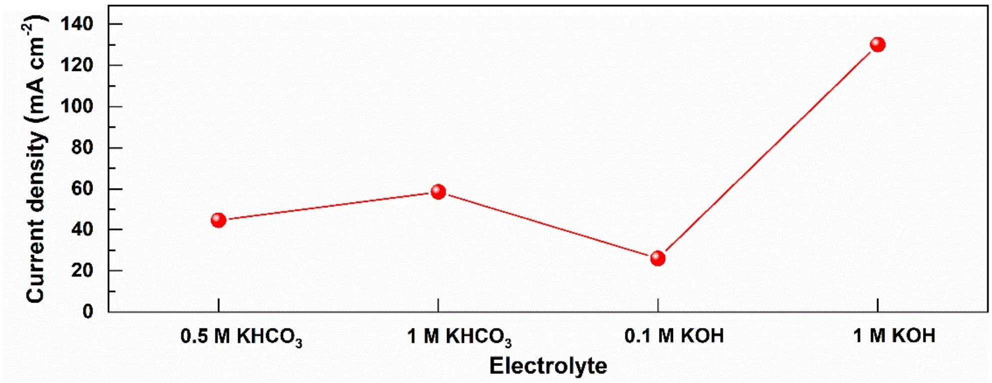


**Supplementary Figure 6.** Total current density using TCNQ@HKUST-1-p/CP electrode for $CO_2$RR in different electrolytes at −2.27 V vs RHE.

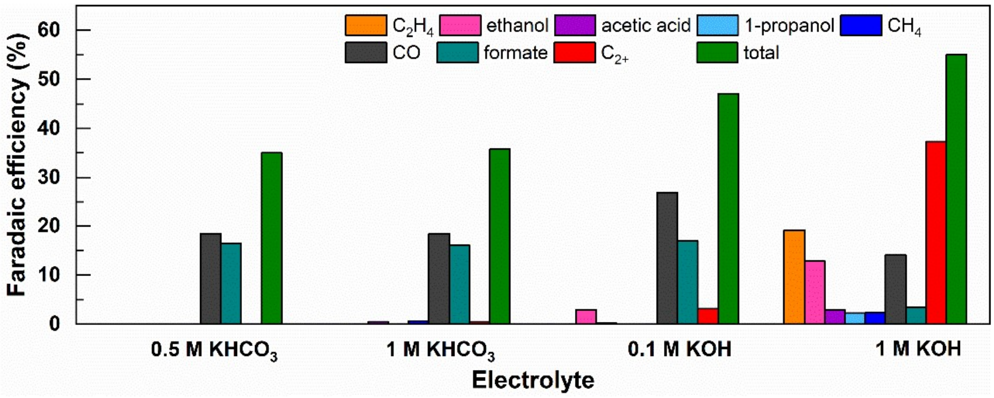


**Supplementary Figure 7.** Catalytic performance of TCNQ@CuMOP-p/CP electrode for $CO_2$RR in different electrolytes at −2.27 V vs RHE.

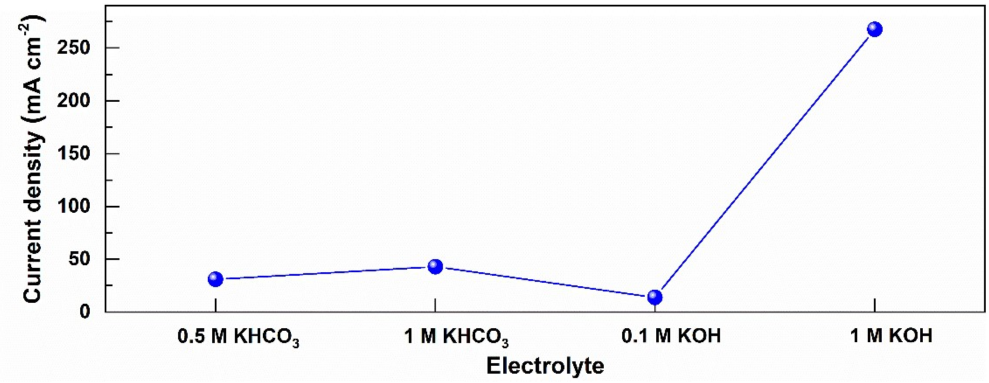


**Supplementary Figure 8.** Total current density using TCNQ@CuMOP-p/CP electrode for $CO_2$RR in different electrolytes at −2.27 V vs RHE.

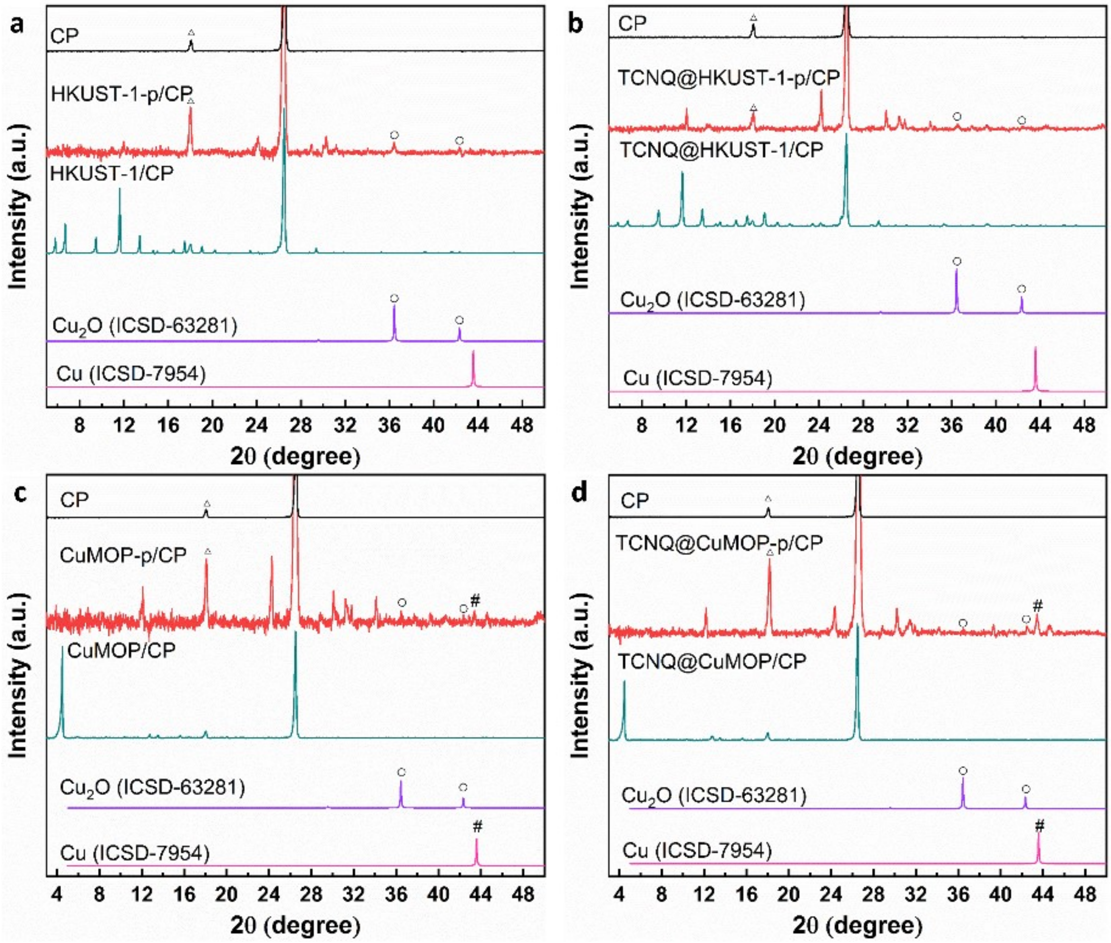


**Supplementary Figure 9.** PXRD patterns of working electrodes before and after 10 mins of electrolysis of $CO_2$.

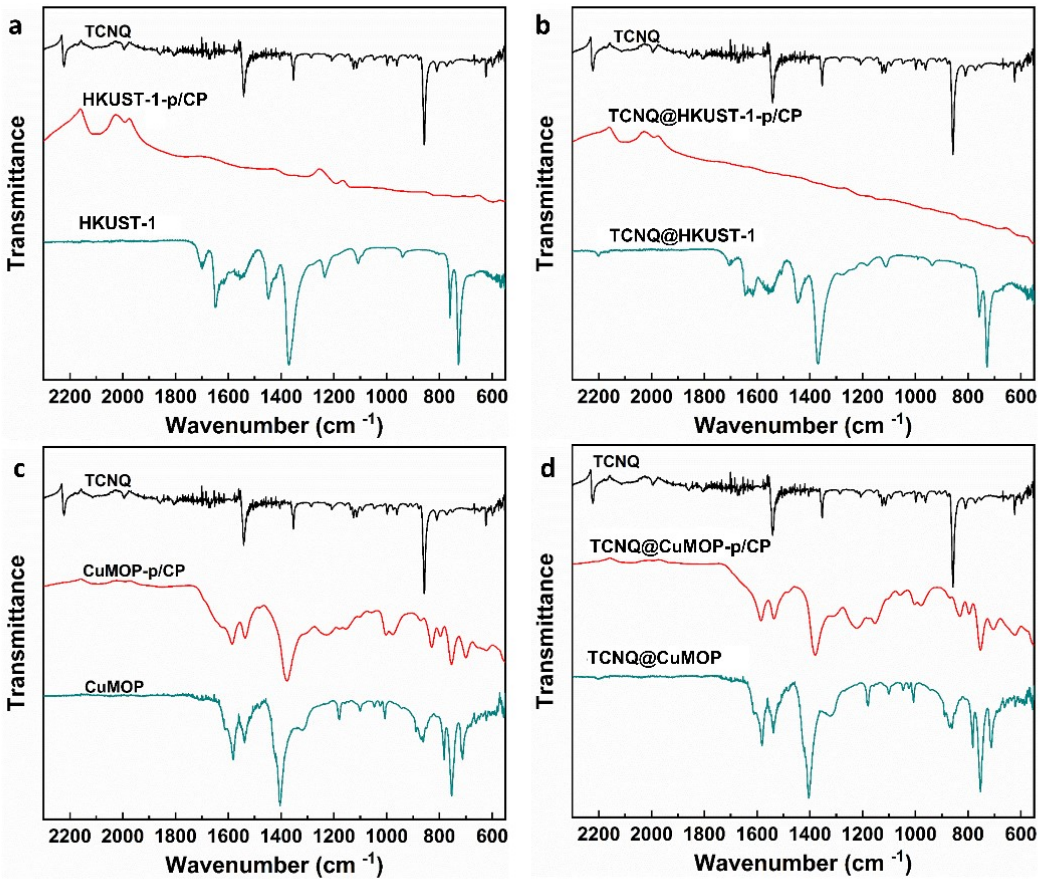


**Supplementary Figure 10.** FTIR spectra of working electrodes before and after 10 mins of

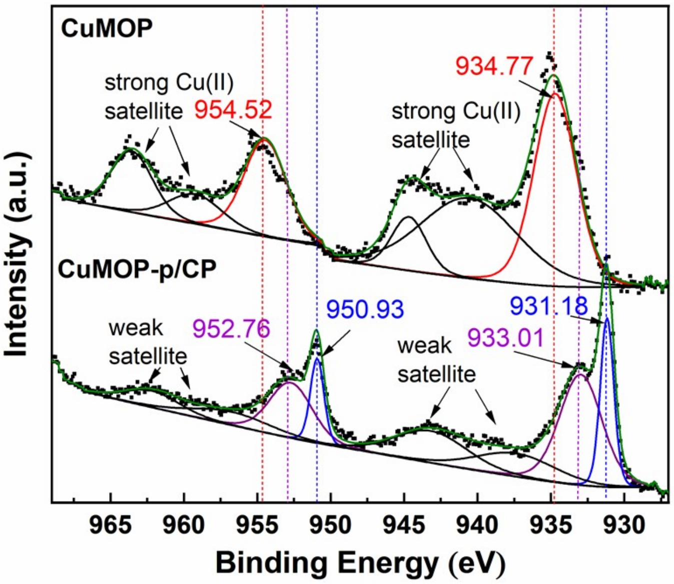


electrolysis of $CO_2$.

**Supplementary Figure 11.** Cu 2p XPS spectra of CuMOP and CuMOP-p/CP.

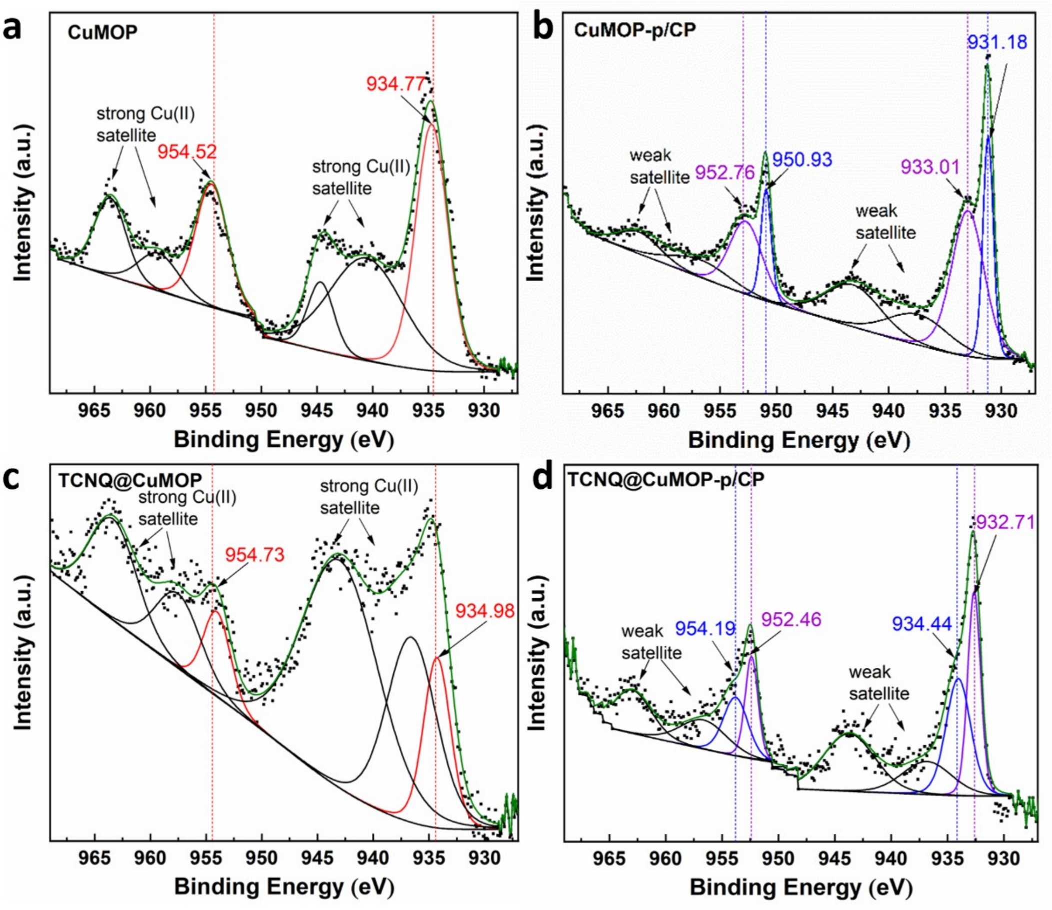


**Supplementary Figure 12.** Cu 2p XPS spectra of working electrodes before and after 10 mins of electrolysis of $CO_2$.

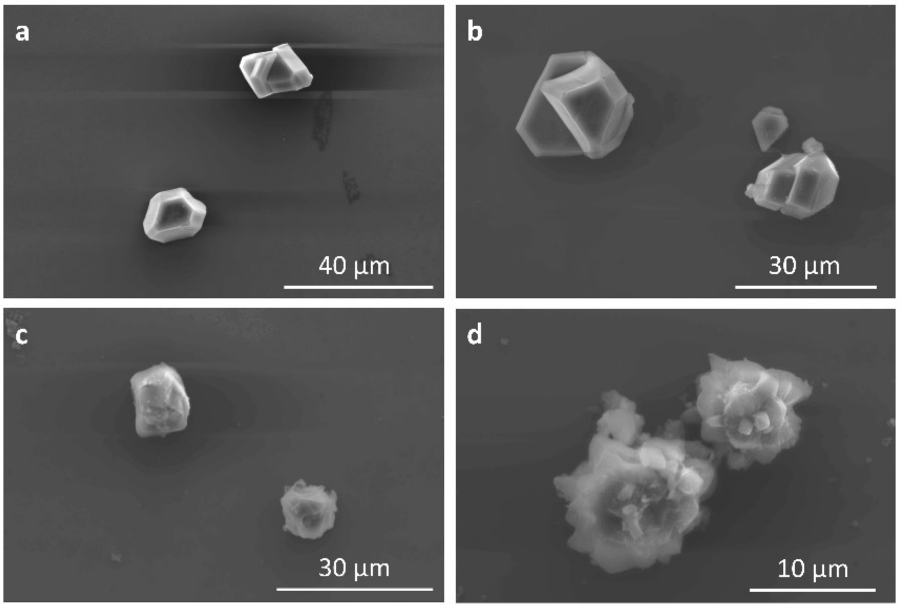


**Supplementary Figure 13.** SEM images. (a) HKUST-1, (b) TCNQ@HKUST-1, (c) CuMOP and (d) TCNQ@CuMOP.

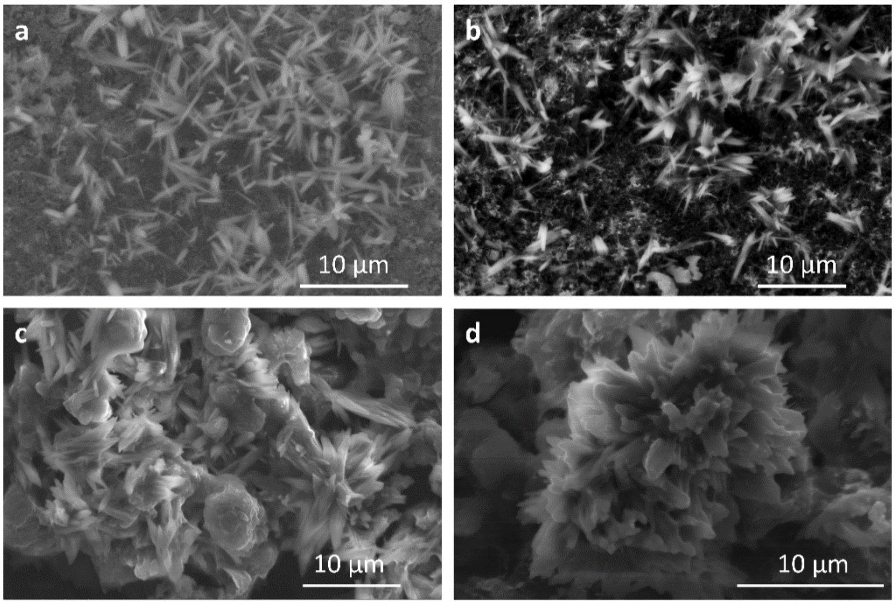


**Supplementary Figure 14.** SEM images. (a) HKUST-1-p/CP, (b) TCNQ@HKUST-1-p/CP, (c) CuMOP-p/CP and (d) TCNQ@CuMOP-p/CP.

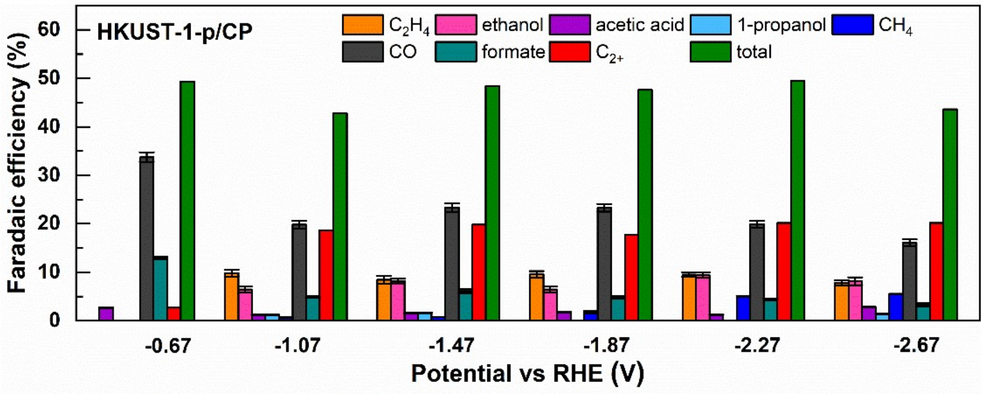


**Supplementary Figure 15.** Catalytic performance of HKUST-1-p/CP electrode for $CO_2RR$ at different potentials.

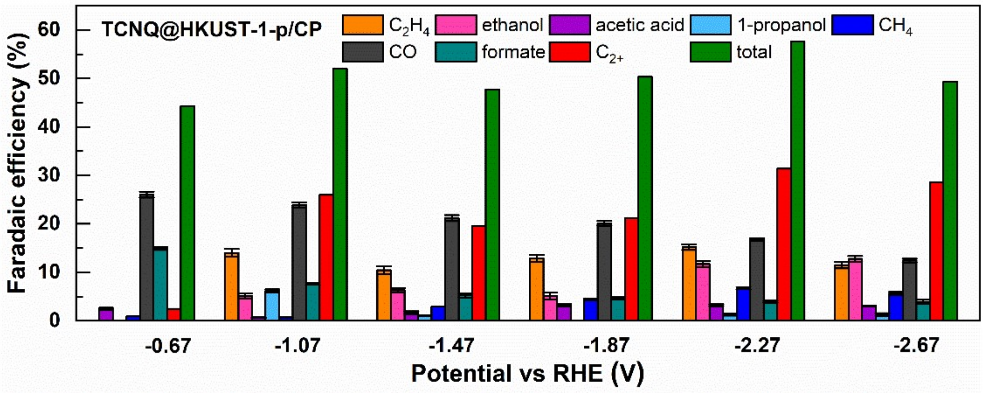


**Supplementary Figure 16.** Catalytic performance of TCNQ@HKUST-1-p/CP electrode for $CO_2RR$ at different potentials.

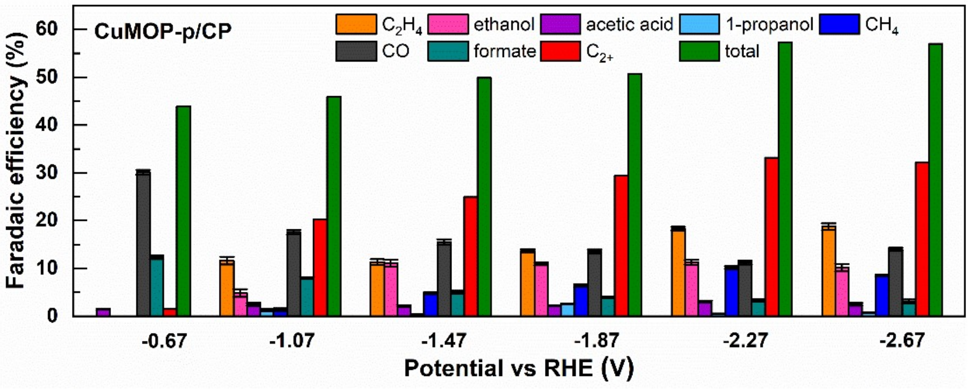


**Supplementary Figure 17.** Catalytic performance of CuMOP-p/CP electrode for $CO_2RR$ at different potentials.

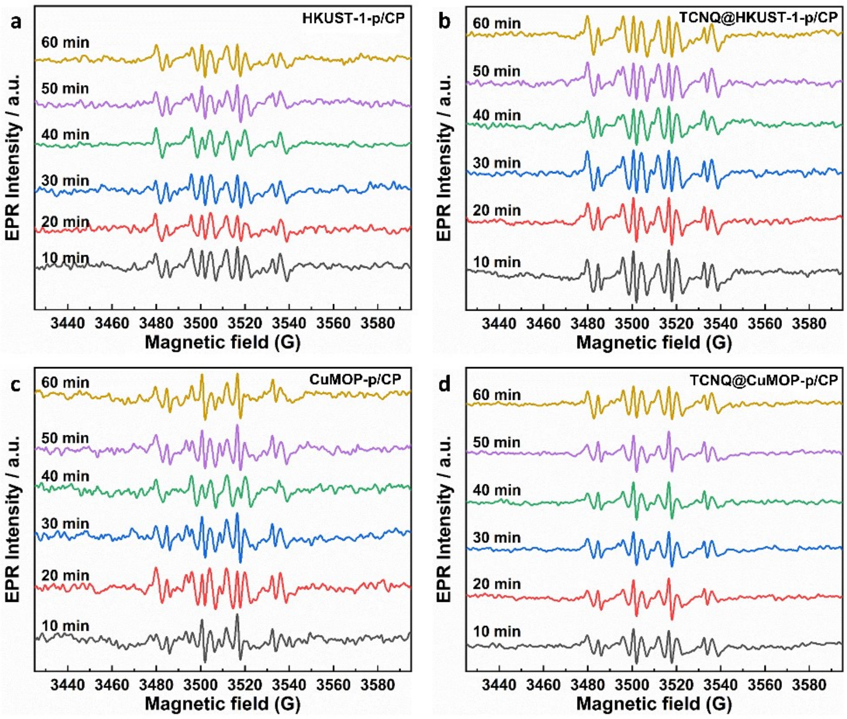


**Supplementary Figure 18.** X-band EPR spectra of electrolyte aliquots taken as a function of time.

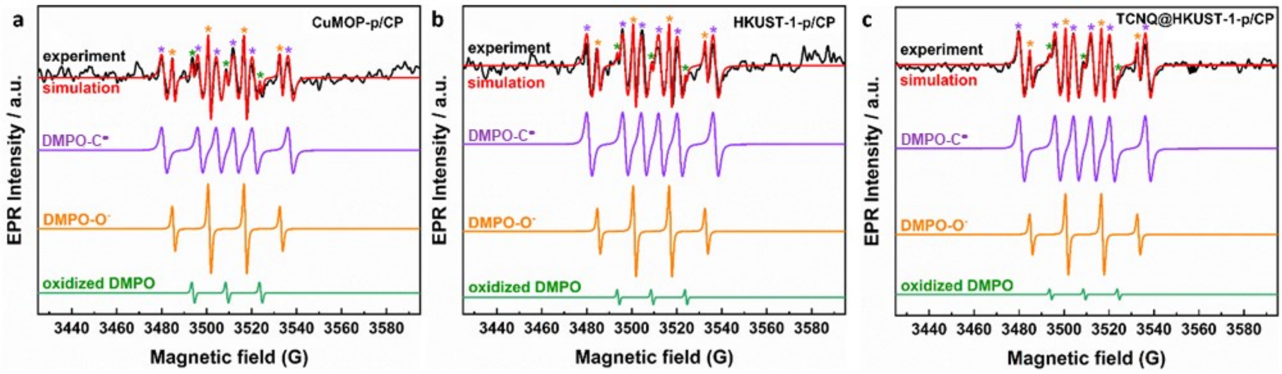


**Supplementary Figure 19.** X-band EPR spectra of spin adducts of free radicals. The complete set of parameters for simulations are given in Supplementary Table 2. Based on the electrochemical $CO_2$ reduction results, most of the products are multi-carbon products, denoted DMPO-C• above. The observed EPR simulated parameters for DMPO carbon-centred radicals are different to those derived from •$CO_2$ and •COOH radicals. In addition, using DMPO as a spin trap does not differentiate between various carbon-centred radicals.

**Supplementary Table 1.** Spin-Hamiltonian parameter set extracted from Q-band CW EPR spectra of as-synthesised HKUST-1, TCNQ@HKUST-1, CuMOP, TCNQ@CuMOP.

| | feature A: Monomeric Cu(II) | feature B: Intra-nuclear exchange in $\{Cu(II)_2\}$ paddlewheels | feature C: Interdinuclear exchange between neighbouring $\{Cu(II)_2\}$ paddlewheels |
|---|---|---|---|
| HKUST-1 | g = [2.069, 2.35]<br>A = [30 MHz, 450 MHz] | g = [2.06, 2.35]<br>D = -0.325 $cm^{-1}$<br>E = 0 $cm^{-1}$ | g = [2.05, 2.30]<br>*J'* = 1 $cm^{-1}$ |
| TCNQ@HKUST-1 | g = [2.090, 2.35]<br>A = [30 MHz, 450 MHz] | - | g = [2.15, 2.35]<br>*J'* = 1 $cm^{-1}$ |
| CuMOP | g = [2.069, 2.35]<br>A = [30 MHz, 450 MHz] | g = [2.06, 2.35]<br>D = -0.325 $cm^{-1}$<br>E = 0 $cm^{-1}$ | g = [2.07, 2.30]<br>*J'* = 1 $cm^{-1}$ |
| TCNQ@CuMOP | g = [2.069, 2.35]<br>A = [30 MHz, 450 MHz] | g = [2.03, 2.2]<br>D = -0.325 $cm^{-1}$<br>E = 0 $cm^{-1}$ | g = [2.05, 2.10]<br>*J'* = 1 $cm^{-1}$ |

*g* and *A* are the *g*-value and Cu hyperfine coupling constants, respectively; *D* and *E* are the axial and rhombic zero-field splitting parameters of the *S* = 1 state, respectively. *J'* = the exchange coupling constant. The simulation of the inter-binuclear exchange line follows previous research in which only one inter-binuclear exchange pathway with an exchange coupling constant *J'* was considered.[3]

**Supplementary Table 2**. EPR spectra simulation parameters of the radicals produced during $CO_2RR$.

| Working electrode | Adduct | g-factor | $A\,^{14}N$ / G | $A\,^{1}H$ / G | lw/mT |
|---|---|---|---|---|---|
| HKUST-1-p/CP | DMPO-C• | 2.0054 | 16.0 | 24.3 | 0.2<br>0.2 |
| | DMPO-O• | 2.0054 | 16.0 | 16.0 | 0.12<br>0.09 |
| | Oxidised DMPO | 2.0055 | 15.1 | - | 0.12 |
| TCNQ@HKUST-1-p/CP | DMPO-C• | 2.0054 | 16.0 | 24.3 | 0.2<br>0.2 |
| | DMPO-O• | 2.0054 | 16.0 | 16.0 | 0.12<br>0.09 |
| | Oxidised DMPO | 2.0055 | 15.1 | - | 0.12 |
| CuMOP-p/CP | DMPO-C• | 2.0054 | 16.0 | 24.3 | 0.2<br>0.2 |
| | DMPO-O• | 2.0054 | 16.0 | 16.0 | 0.12<br>0.09 |
| | Oxidised DMPO | 2.0055 | 15.1 | - | 0.12 |
| TCNQ@CuMOP-p/CP | DMPO-C• | 2.0054 | 16.0 | 24.3 | 0.2<br>0.2 |
| | DMPO-O• | 2.0054 | 16.0 | 16.0 | 0.12<br>0.09 |
| | Oxidised DMPO | 2.0055 | 15.1 | - | 0.12 |

* lw is the homogeneous Lorentzian linewidth; *g* and *A* are the g and hyperfine constant parameters, respectively. The spin trap DMPO can be oxidized by $O_2$ during the EPR measurement. Therefore, it is normal to detect oxidised DMPO in such experiments.

**Supplementary Table 3.** The performance of Cu(II)-based MOFs and their derivatives for $CO_2$RR.

| **Catalyst** | **Pre-processing method** | **Main product** | **Electrolyte** | **Faradaic efficiency (%)** | **Potential V vs RHE** | **Current density mA·cm$^{-2}$** | **Ref** |
|---|---|---|---|---|---|---|---|
| $Cu_2O$@Cu-MOF | - | $CH_4$ | 0.1 M $KHCO_3$ | 63 | -1.71 | 8.4 | 4 |
| Cu-THQ | - | CO | 1 M $C_5H_{14}ClNO$ + 1 M KOH | 91 | −0.45 | 173 | 5 |
| d-Cu-1 derivatives | Electro-reduction | formate | IL/MeCN/$H_2O$ | 98 | −1.85 vs Ag/Ag+ | 102.1 | 6 |
| S-HKUST-1 | Guest introducing | $C_2H_4$ | 1 M KOH | 57 | NA | 400 | 7 |
| Cu NPs from Cu-MOF-74 | Electro-reduction | $CH_4$ | 0.1 M $KHCO_3$ | 50 | −1.3 | 10 | 8 |
| Cu-DBC | - | $CH_4$ | 0.1 M $KHCO_3$ | 56 | −1.4 | 11.4 | 9 |
| MAF-2E | - | $CH_4$ + $C_2H_4$ | 0.1 M $KHCO_3$ | 77 | −1.5 | ~17 | 10 |
| Cu−MOF/NP | Calcination | CO | 0.5 M $KHCO_3$ | 44 | −0.86 | 230 | 11 |
| H-CuTCPP@$Cu(OH)_2$ | - | acetic acid | 0.5 M $EMIMBF_4$ MeCN (1 M $H_2O$) | 26 | −1.6 vs. Ag/Ag+ | ~7.5 | 12 |
| HATNA-Cu-MOF | - | $CH_4$ | 0.1 M $KHCO_3$ | 78 | −1.5 | 8.2 | 13 |
| HKUST@800 | Calcination | $C_{2+}$ product | 1 M KOH | 54 | NA | 80 | 14 |
| $Cu_4$-MFU-4l | - | $CH_4$ | 0.5 M $NaHCO_3$ | 88 | −1.3 | 18.3 | 15 |
| H-265 | Calcination | $C_2H_4$ | 1 M KOH | 51 | −1.58 | 150 | 16 |
| CPFs | - | $CH_4$ + $C_2H_4$ | 0.1 M $KHCO_3$ | 74 | −1.4 | 7.5 | 17 |
| Cu(111)@Cu-THQ | Electro-reduction | $C_2H_4$ | 0.1 M $KHCO_3$ | 42 | −1.4 | 14.3 | 18 |
| $Cu_2O$/Cu@NC-800 | Calcination | formate | 0.1 M $KHCO_3$ | 71 | −0.68 | 4.4 | 19 |
| $Cu_2O$@CuHHTP | Electro-reduction | $CH_4$ | 0.1 M KCl/0.1 M $KHCO_3$ | 73 | −1.4 | 10.8 | 20 |
| OD-Cu-3 | Calcination | $C_{2+}$ product | 1 M KOH | 70 | −1.3 | 141 | 21 |
| HKUST-1-p | Electro-reduction | $C_{2+}$ product | 1 M KOH | 37 | −2.27 | 100 | THIS WORK |
| TCNQ@HKUST-1-p | Electro-reduction | $C_{2+}$ product | 1 M KOH | 33 | −2.27 | 52.2 | THIS WORK |
| CuMOP-p | Electro-reduction | $C_{2+}$ product | 1 M KOH | 31 | −2.27 | 40.9 | THIS WORK |
| TCNQ@CuMOP-p | Electro-reduction | $C_{2+}$ product | 1 M KOH | 20 | −2.27 | 24.0 | THIS WORK |

**References**


1. S. P. Argent, I. da Silva, A. Greenaway, M. Savage, J. Humby, A. J. Davies, H. Nowell, W. Lewis, P. Manuel, C. C. Tang, A. J. Blake, M. W. George, A. V. Markevich, E. Besley, S. Yang, N. R. Champness and M. Schröder, *Inorg. Chem.*, 2020, **59**, 15646-15658.
2. S. Stoll and R. D. Britt, *Phys. Chem. Chem. Phys*, 2009, **11**, 6614-6625.
3. M. Šimėnas, M. Kobalz, M. Mendt, P. Eckold, H. Krautscheid, J. r. Banys and A. Pöppl, *J. Phys. Chem. C*, 2015, **119**, 4898-4907.

4. X. Tan, C. Yu, C. Zhao, H. Huang, X. Yao, X. Han, W. Guo, S. Cui, H. Huang and J. Qiu, *ACS Appl. Mater. Interfaces*, 2019, **11**, 9904-9910.
5. L. Majidi, A. Ahmadiparidari, N. Shan, S. N. Misal, K. Kumar, Z. Huang, S. Rastegar, Z. Hemmat, X. Zou and P. Zapol, *Adv. Mater.*, 2021, **33**, 2004393.
6. Q. Zhu, D. Yang, H. Liu, X. Sun, C. Chen, J. Bi, J. Liu, H. Wu and B. Han, *Angew. Chem. Int. Ed.,* 2020, **132**, 8981-8986.
7. C. F. Wen, M. Zhou, P. F. Liu, Y. Liu, X. Wu, F. Mao, S. Dai, B. Xu, X. L. Wang, Z. Jiang, P. Hu, S. Yang, H. F. Wang and H. G. Yang, *Angew. Chem. Int. Ed.*, 2022, **61**, e202111700 (1-8).
8. M. K. Kim, H. J. Kim, H. Lim, Y. Kwon and H. M. Jeong, *Electrochim. Acta*, 2019, **306**, 28-34.
9. Y.-Y. Liu, H.-L. Zhu, Z.-H. Zhao, N.-Y. Huang, P.-Q. Liao and X.-M. Chen, *ACS Catal.*, 2022, **12**, 2749-2755.
10. L. L. Zhuo, P. Chen, K. Zheng, X. W. Zhang, J. X. Wu, D. Y. Lin, S. Y. Liu, Z. S. Wang, J. Y. Liu and D. D. Zhou, *Angew. Chem. Int. Ed.*, 2022, **134**, e202204967 (1-5).
11. J. Liu, L. Peng, Y. Zhou, L. Lv, J. Fu, J. Lin, D. Guay and J. Qiao, *ACS Sustain. Chem. Eng.*, 2019, **7**, 15739-15746.
12. Y.-H. Xiao, Y.-X. Zhang, R. Zhai, Z.-G. Gu and J. Zhang, *Sci. China Mater.* 2022, **65**, 1269-1275.
13. Y. Liu, S. Li, L. Dai, J. Li, J. Lv, Z. Zhu, A. Yin, P. Li and B. Wang, *Angew. Chem. Int. Ed.*, 2021, **133**, 16545-16551.
14. N. Sikdar, J. R. Junqueira, S. Dieckhöfer, T. Quast, M. Braun, Y. Song, H. B. Aiyappa, S. Seisel, J. Weidner and D. Öhl, *Angew. Chem. Int. Ed.*, 2021, **60**, 23427-23434.
15. H.-L. Zhu, J.-R. Huang, X.-W. Zhang, C. Wang, N.-Y. Huang, P.-Q. Liao and X.-M. Chen, *ACS Catal.*, 2021, **11**, 11786-11792.
16. K. Yao, Y. Xia, J. Li, N. Wang, J. Han, C. Gao, M. Han, G. Shen, Y. Liu and A. Seifitokaldani, *J. Mater. Chem. A*, 2020, **8**, 11117-11123.
17. Y. Zhou, S. Chen, S. Xi, Z. Wang, P. Deng, F. Yang, Y. Han, Y. Pang and B. Y. Xia, *Cell Rep. Phy. Sci.*, 2020, **1**, 100182.
18. Z.-H. Zhao, K. Zheng, N.-Y. Huang, H.-L. Zhu, J.-R. Huang, P.-Q. Liao and X.-M. Chen, *Chem. Commun.*, 2021, **57**, 12764-12767.
19. D. Li, T. Liu, Z. Yan, L. Zhen, J. Liu, J. Wu and Y. Feng, *ACS Appl. Mater. Interfaces*, 2020, **12**, 7030-7037.
20. J. D. Yi, R. Xie, Z. L. Xie, G. L. Chai, T. F. Liu, R. P. Chen, Y. B. Huang and R. Cao, *Angew. Chem. Int. Ed.*, 2020, **59**, 23641-23648.
21. F. Yang, P. Deng, Q. Wang, J. Zhu, Y. Yan, L. Zhou, K. Qi, H. Liu, H. S. Park and B. Y. Xia, *J. Mater. Chem. A*, 2020, **8**, 12418-12423.
